# The effects of information technology interventions for optimizing antibiotic prescribing in urinary tract infections: a systematic review

**DOI:** 10.1186/s12879-025-12170-0

**Published:** 2025-12-29

**Authors:** Fatemeh Rangraz Jeddi, Ehsan Nabovati, Reihane Sharif, Amin Sadat Sharif, Shima Anvari

**Affiliations:** 1 Health Information Management Research Center, Kashan University of Medical Sciences, Kashan, Iran; 2 Shahid Hasheminejad Hospital, Iran University of Medical Sciences, Tehran, Iran

**Keywords:** Urinary tract infections, Antibiotic prescription, Antimicrobial resistance, Information technology

## Abstract

**Objectives:**

Information technology (IT) solutions can facilitate evidence-based decision-making for antibiotic use by delivering timely information directly to clinicians at the point of care. This study aimed to evaluate the effects of IT interventions in optimizing antibiotic prescribing for urinary tract infections (UTIs).

**Methods:**

A comprehensive search was performed in Medline (through PubMed), Web of Science, and Scopus databases from inception to June 2024. The study included randomized controlled trials (RCTs) and cluster randomized controlled trials (CRCTs) that investigated the effects of IT interventions on optimizing antibiotic prescribing for UTI patients. Participants were patients with UTI. IT interventions were used for improving antibiotic prescribing. Two researchers independently extracted data from studies on study characteristics, intervention details, and intended outcomes.

**Results:**

Ten eligible studies (5 RCTs and 5 CRCTs) were included. Clinical decision support systems (CDSS) were the most common intervention type (50% of studies), often integrated with electronic health records (EHRs). Results showed mixed effects across patient-related, prescriber-related, and economic outcomes. While patient outcomes, typically measured as secondary endpoints, showed no statistically significant differences, prescriber-related outcomes showed more promising results. Four studies reported significant reductions in overall antibiotic prescribing rates, and two studies demonstrated significant increases in antibiotic appropriateness. Additionally, some studies showed decreased laboratory test orders and reduced emergency department visit durations. However, economic outcomes remained largely unaffected.

**Conclusion:**

The review suggests that IT interventions, especially those focusing on prescriber behavior, hold significant promise for optimizing antibiotic prescribing practices in UTI management. However, further research is needed to explore their effect on patient outcomes and cost-effectiveness. The findings underscore the potential of IT interventions as valuable tools for combating antibiotic resistance in UTI treatment.

**Clinical trial:**

Not applicable.

**Supplementary Information:**

The online version contains supplementary material available at 10.1186/s12879-025-12170-0.

## Background

Urinary tract infections (UTIs) are prevalent across outpatient and inpatient healthcare settings [1]. Physicians differentiate UTIs into two primary categories: uncomplicated and complicated. Uncomplicated UTIs generally affect healthy individuals with no underlying structural or neurological abnormalities in the urinary tract [2, 3]. These infections can be further subclassified as lower UTIs involving the bladder (cystitis) or upper UTIs affecting the ureters and kidneys (pyelonephritis) [3, 4]. Complicated UTIs are associated with predisposing factors such as urinary tract obstructions, neurogenic bladder dysfunction, immunosuppression, renal insufficiency, kidney transplantation, pregnancy, or the presence of foreign bodies, such as indwelling urinary catheters, ureteral stents, and nephrostomy tubes [5, 6].

While antibiotics are the primary treatment for UTIs, effectively relieving symptoms and preventing recurrences, challenges in accurate diagnosis often lead to inappropriate prescribing [7, 8]. This practice increases the risk of antimicrobial resistance (AMR) among patients [9] but also unnecessarily exposes patients to potential adverse effects of antibiotics, including allergic reactions, end-organ toxic effects, subsequent infection with antibiotic-resistant organisms, and clostridium difficile infections (CDIs) [10]. To combat AMR, judicious antibiotic prescribing is crucial. This encompasses two key components: first, prescribing antibiotics only when clinically indicated; and second, when indicated, selecting the appropriate antibiotic based on clinical presentation, using the correct dosage, and prescribing the optimal treatment duration [11].

Clinical practice guidelines (CPGs), such as those from the Infectious Diseases Society of America (IDSA) and the European Association of Urology (EAU), offer evidence-based recommendations for antibiotic choice, dosing, and duration in UTI treatment [12, 13]. They stress tailoring therapy to local resistance patterns, patient factors, and infection severity. However, studies have demonstrated poor guideline adherence among healthcare providers, for UTIs in various settings. This non-adherence contributes significantly to the development and spread of antimicrobial resistance by promoting the selection of resistant bacterial strains through suboptimal antibiotic use [14, 15]. Addressing inappropriate antibiotic prescribing in UTIs requires a multifaceted approach using strategies that may include education and training, audit and feedback, physician reminders, and point-of-care diagnostic testing solutions [16].

Information technology (IT) interventions can significantly enhance the implementation of effective antibiotic stewardship strategies [17, 18]. Key IT tools include computerized provider order entry (CPOE), clinical decision support systems (CDSS), computerized antimicrobial approval systems (CAAS), and surveillance systems (SSs) which can be integrated into electronic health records (EHRs) to provide physicians with timely and accurate information at the point of care, facilitating more informed decision-making regarding antibiotic prescribing [19, 20].

Existing systematic review on antibiotic prescribing for UTIs, have primarily focused on traditional non-IT interventions (such as audit and feedback, and reminder systems), targeting general practitioners (GPs) in primary care settings [21]. However, this review has often overlooked the potential of IT-based interventions in optimizing antibiotic use. Additionally, another systematic review has been limited by a narrow focus on specific IT tools, such as CDSS, with a primary focus on evaluating urine culture test rates [22]. The present study was conducted with the aim of investigating the effect of IT-based interventions on prescription of antibiotics to patients with UTI in all age groups and in all medical centers (including primary and secondary) until 2024.

## Materials and methods

### Data sources and search strategy

This systematic review followed the Preferred Reporting Items for Systematic Reviews and Meta-Analyses (PRISMA) guidelines [23]. Three major medical electronic databases, Medline (via PubMed), ISI Web of Science, and Scopus, were systematically searched using a predefined search strategy. This strategy incorporated a combination of Medical Subject Headings (MeSH) terms and keywords relevant to the three core domains of interest: UTI, antibiotic prescribing practices, and IT (Appendix 1). The search strategy was peer-reviewed using the Peer Review of Electronic Search Strategies (PRESS) Checklist [24] before execution. The search encompassed all studies published from database inception to June 2024. Retrieved records from all databases were imported into EndNote reference management software (version X9), and duplicate records were identified and removed using both automated and manual screening processes to ensure no study was evaluated more than once. To ensure comprehensive coverage, reference lists of identified studies and relevant existing systematic reviews were hand-searched for additional pertinent papers.

### Inclusion and exclusion criteria

Studies were eligible for inclusion if they met the following criteria: (a) UTIs were the primary focus of the study, with antibiotic prescribing outcomes for patients with UTI were reported separately; (b) IT interventions employed to improve these clinical practices; (c) studies with intervention and control groups where outcomes related to antibiotic prescribing were compared between the groups; (d) the primary outcome was the rate of antibiotic prescription. Additionally, other outcomes relevant to improved antibiotic prescribing were considered; (e) the study design was randomized controlled trials (RCTs) or cluster randomized controlled trials (CRCTs).

Studies were excluded based on the following criteria: (a) UTIs were not the primary focus of the study, or outcomes for UTI patients could not be separately identified in studies examining multiple infection types (b) interventions implemented for improving antibiotic prescribing did not utilize IT strategies as the main intervention; (c) studies lacking data on antibiotic prescribing outcomes in both intervention and control groups; (d) studies focused on prescribing medications other than antibiotics; (e) review articles, letters to the editor, protocols, conference presentations, and dissertations/theses.

### Screening and data extraction

A standardized data extraction form was developed to ensure consistency in data collection. Two independent reviewers (Sh.A and R.Sh) screened titles, abstracts, and full texts of retrieved studies based on the predefined eligibility criteria. Data extraction was performed independently by the same two reviewers using a standardized data extraction form. Any discrepancies in extracted data were resolved through discussion until consensus was reached, and a third reviewer (E.N) was consulted when necessary. Inter-rater agreement for study screening and data extraction was assessed using Cohen’s Kappa, yielding excellent reliability with a Kappa value of 0.9. From each study, the following data were extracted: the study characteristics (including publication year, country, setting, design, sample size, and participations’ age), the intervention characteristics, and the study’s intended outcomes.

### Data synthesis and analysis

Due to the potential challenges of harmonizing data from cluster RCTs (CRCTs) and the diversity observed in the included studies (variation in interventions, target populations, and study settings), a meta-analysis was not feasible. Consequently, the findings were presented through a narrative synthesis of the available evidence. To facilitate a structured analysis, the included studies were categorized based on several key characteristics: characteristics of interventions, outcome classification, and intervention effects on outcomes. The effect of interventions on outcomes was categorized into established categories employed in similar systematic review [25]: positive effect (statistically significant), positive effect (not stated about significance) and not statistically significant.

### Study quality assessment

The Cochrane Collaboration’s assessment tool was employed to evaluate potential bias in the selected studies. This tool, recognized for its validity and reliability in examining RCTs, focuses on six key areas: Selection, Performance, Detection, Attrition, Reporting, and Other Biases [26]. Each area was rated as high, low, or unclear risk. During the data extraction process, two reviewers (Sh.A and R.Sh) independently evaluated the studies. Any disagreements were resolved through discussion with a third reviewer.

## Results

### Literature search results

**Fig. 1:**
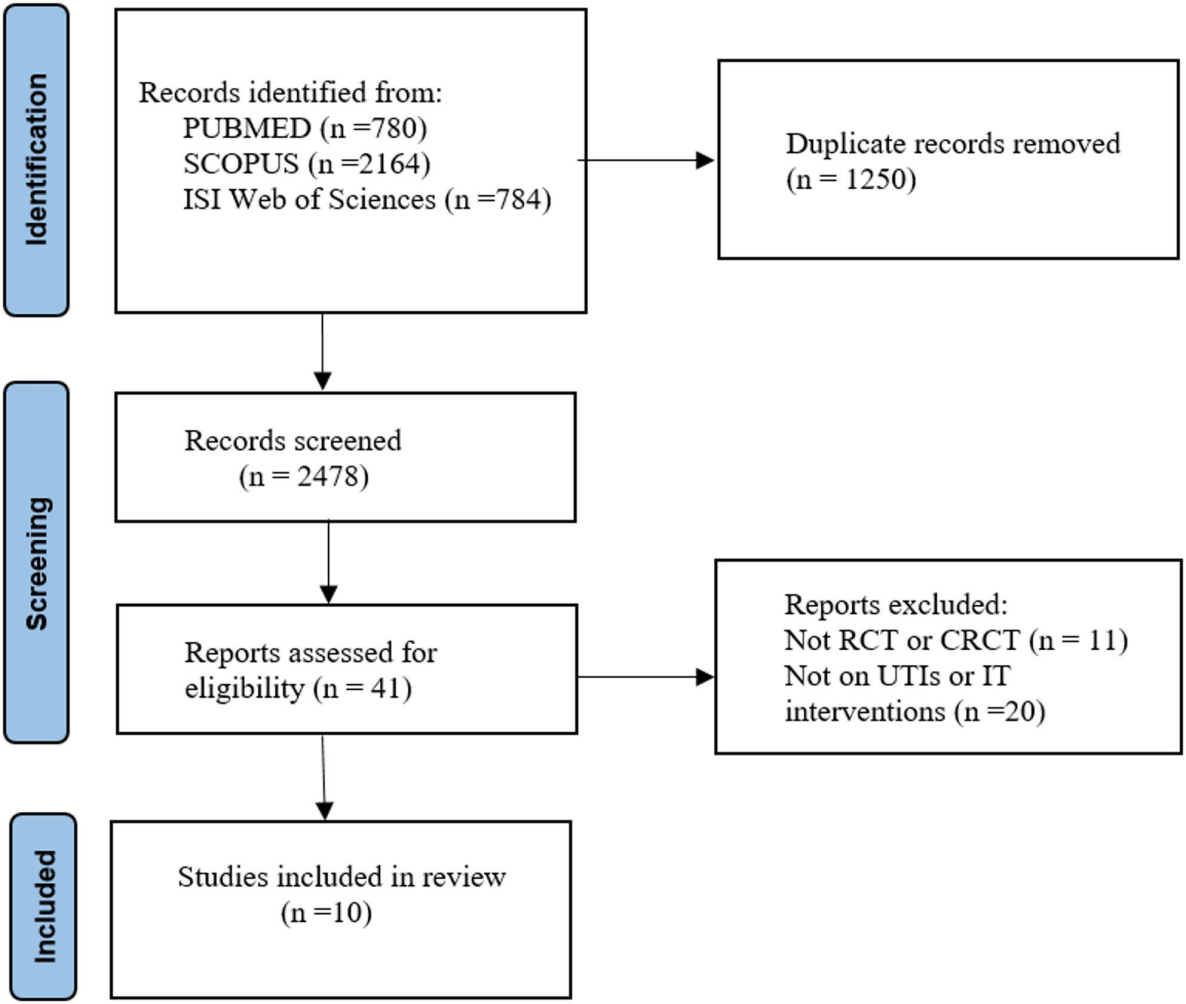
Flow diagram of the literature and study selection

The search results and study selection process are summarized in Fig. 1. In total, 3728 studies were found. After removing 1250 duplicate records, 41 studies were selected for full-text review following title and abstract screening. Of these, 31 studies were excluded for the following reasons: inappropriate study design (*n* = 11), non-IT interventions (*n* = 10), and not focused on UTI (*n* = 10).

## Characteristics of the included studies

The characteristics of the included studies are presented in Table 1. The systematic review included ten studies that evaluated the effect of IT-based interventions on antibiotic prescribing for UTIs. These studies employed rigorous research designs, including five RCTs and five CRCTs. All studies were conducted in high-income countries, with five in the United States and five in Europe. The interventions were implemented in a variety of healthcare settings, including primary care, emergency departments, hospitals, and nursing homes. The studies varied widely in terms of patient’s sample size (range: 72-6849), patients age (mean age range: 31.4–86.3 years), and intervention duration (range: 7 days-36 months). Two studies specifically focused on older adult populations.

**Table 1:** Main characteristics of the included studies (ranked according to year of publication)

Study	Year	Country	Setting	Study Design	Sample Size	Mean Age (year)	Duration	Intervention Type	
								IT	Others
[27]	2001	United States	6 GPs	RCT	72 women with suspected UTI	36.6	7 days	Telephone	-
[28]	2001	Norway	142 GPs	CRCT	5734 women with UTI	36.2	8 months	CDSS, reminders, Telephone consultations	Patient educational material, Interactive courses
[29]	2008–2010	United States	3 EDs	RCT	103 women with suspected UTI	31.4	20 months	Computer kiosk	-
[30]	2012	United States	Single urban ED	RCT	200 adults with UTI (Men and women)	33	4 months	SMS query, phone follow-up	-
[31]	2013–2014	Ireland	30 GPs‎	CRCT	920 women with suspected UTI	56.1	9–10 months	Reminder pop-up, Multimedia application	Coding workshop, interactive workshop, Audit reports
[32]	2015–2018	United States	4 North Shore hospitals	RCT	6849 women with UTI	71.06	36 months	CDSS (WISCA tool)	-
[33]	2019	United States	2 VA medical centers	RCT	272 afebrile men with presumed symptomatic UTI	69	14 days	Telephone visits	Antibiotic prescription
[34]	2019–2021	Multiple European countries	43 GPs‎ and 43 older adult care organizations	CRCT	1041 frail older adults (Men and women)	86.3	9 months	Decision tool and e-learning module, (video)	Participatory action research
[35]	2020	Netherlands	16 Nursing homes	CRCT	212 residents with suspected UTI (Men and women)	86	12 months	EHR-integrated decision tool,	Training, Pocket-cards
[36]	2021–2022	Germany	110 GPs	CRCT	203 patients with suspected UTI (Men and women)	52	12 months	Telephone counselling	Guideline recommendations, feedback

IT: Information Technology (primary intervention), Others: Additional non-IT interventions used in combination with IT interventions, GPs: General practices, ED: Emergency Department, VA: Veterans Affairs, RCT: Randomized Controlled Trial, UTI: Urinary Tract Infection, CRCT: Cluster Randomized Trials, CDSS: Clinical Decision Support Systems, SMS: Short Message Service, WISCA: ‎ Weighted Incidence Syndromic Combination Antibiogram is an antimicrobial stewardship tool that utilizes electronic medical record data to provide real-time clinical decision support regarding empiric antibiotic prescription in the hospital setting, EHR: Electronic Health Record

### Quality of the included studies

**Fig. 2:**
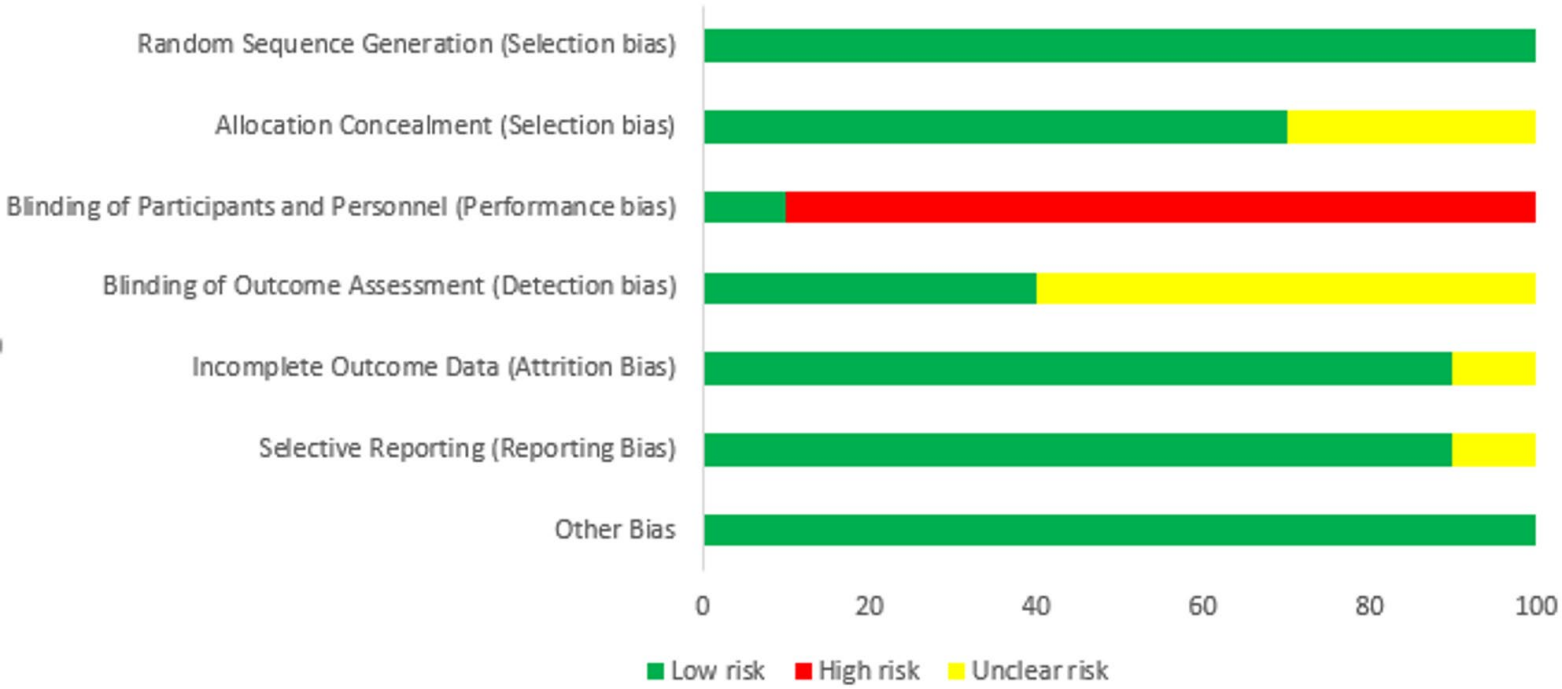
Risk of biases assessment

The results of the quality assessment of the included studies are shown in Fig. 2 and Appendix 2; 100% of the studies specifically reported on their allocation sequence generation and 50% reported allocation sequence concealment. 100% of the studies reported information about the blinding of the participants and the personnel, in 10% of which the blinding was done (low risk) and in the other 90% it was not done (high risk). Moreover, blinding of the outcome assessment was reported as low risk in 40% of the studies. Attrition was also discussed in 90% of the studies, all of which had a low risk of incomplete outcome data. Selective reporting was assessed in all studies based on the match between the predetermined outcomes and the reported outcomes, and 90% of the studies had a low selective reporting bias. Other sources of bias were reported as low risk in 100% of the included studies.

### Description of IT interventions

**Fig. 3:**
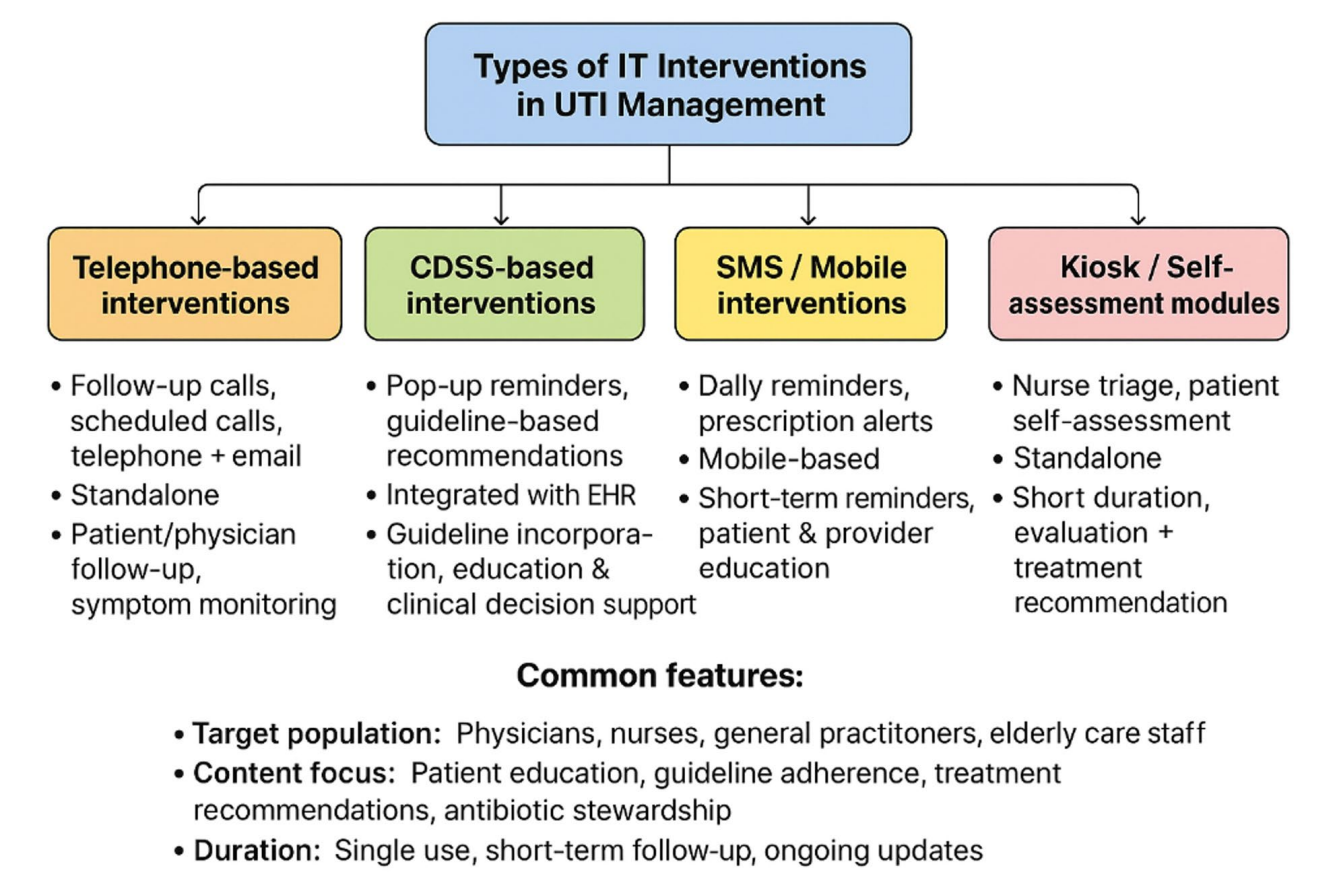
Type of IT interventions in UTI management

The analysis of Table 2 reveals a diverse array of interventions aimed at improving antibiotic prescribing for UTIs. CDSS was the most common intervention type (*n* = 5), followed by telephone consultations (*n* = 3). Other interventions included short message service (SMS) programs, kiosk modules (interactive computer stations where patients self-assess symptoms), and a combination of these. These interventions primarily targeted physicians, GPs, and nurses. The duration of interventions ranged from a few days to several weeks, and the frequency of intervention interaction (i.e., how often clinicians or patients engaged with the IT system) varied from daily to once every three months. The content consistently focused on various aspects of care, such as patient education (*n* = 5), antibiotic stewardship (*n* = 3), and symptom assessment (*n* = 2). Interventions were delivered through various channels, including EHRs, standalone platforms, and mobile devices. Figure 3 visually summarizes the types of interventions and their key features.

**Table 2:** Features of IT interventions in the included studies

Study	Intervention Type	Target Population	Key Features	Duration	Content Focus	Integration^*^
[27]	Telephone	Physicians (not mentioned)	Follow-up calls	Twice daily for 7 days	Patient instructions	Standalone
[28]	CDSS, telephone consultations	General practitioners	Multiple formats	Not specified	Clinical guidelines, Patient education	EHR
[29]	kiosk module, phone follow-up	Nurse triage staff	Self-assessment	5 min use, 2–4week follow-up	UTI evaluation, Treatment recommendations	Standalone
[30]	SMS program	physicians, nurses, or physician extenders	Daily reminders	3 days	Prescription reminders, Education	Mobile
[31]	CDSS	General practitioners	Pop-up reminders	Not specified	Guidelines, Patient education	EHR
[32]	CDSS	Physicians (antimicrobial stewardship)	Antibiotic stewardship	Within 24 h of prescription	Antibiotic recommendations	EHR
[33]	Telephone	Physicians (not mentioned)	Scheduled calls	Not specified	Symptom assessment, Treatment follow-up	Standalone
[34]	CDSS	General practitioners	Guideline-based	Not specified	Antibiotic use guidance, Active monitoring approach	Guideline incorporation
[35]	CDSS	Physicians (elderly care) and nursing staff	Multi-modal education	Not specified	UTI assessment, Treatment guidelines	EHR
[36]	telephone, email	General practitioners	Regular updates	once every three months	Antibiotic stewardship, Resistance data	Standalone

Kiosk modules: refer to standalone computer terminals placed in clinical settings where patients independently complete symptom assessments and receive preliminary guidance, CDSS: Clinical Decision Support System, EHR: Electronic Health Record, SMS: Short Message Service, UTI: Urinary Tract Infection, * Refers to how the IT intervention is incorporated into the workflow or decision-making process for antibiotic prescribing

### Effects of IT interventions on outcomes

Table 3 summarizes the effects of IT interventions across various outcome categories, including patient-related, prescriber-related, and economic outcomes. While patient-related outcomes showed a potential positive association with IT interventions for UTI resolution, statistical significance was not reached. However, no significant improvements were observed in recurrence rates, symptom scores, or overall patient symptomatology. Regarding prescriber-related outcomes, four studies reported statistically significant reductions in overall antibiotic prescribing rates. Additionally, two studies reported statistically significant increases in the appropriateness of prescribed antibiotics. Only one study failed to detect a significant effect on antibiotic use rates. Additionally, two studies demonstrated a significant decrease in the number of laboratory tests and urine cultures ordered, as well as reduced emergency department visit durations following IT intervention implementation. However, the economic category remained unaffected, with no significant decrease observed in antibiotic charges.

**Table 3:** The effects of IT interventions on outcomes

Outcomes			Effect		
			Positive effect (Statistically significant)	Positive effect (Not stated about significance)	Not statistically significant
Patient outcomes	UTI	Resolution		[33]	
		Recurrence			[33]
		Score			[27,]
		Symptoms			[27]
	Antibiotic resistance (acquisition of multidrug-resistant organisms)				[32]
	Adherence rate				[30]
	Time to illness resolution				[29]
	Patient satisfaction				[(27, 29]
	Return visits and phone follow-up				[29, 30]
	LOS				[32]
	Mortality (30-day and all-cause)				[32, 34, 35]
	30-day readmission				[32]
	Clostridium difficile infection within 180 days				[32]
	Adverse events and complications				[33–35]
	Hospital referrals and admissions				[34, 35]
Prescriber outcomes	Antibiotic Prescribing Rates	Changes in rates of antibiotic use			[28]
		Antibiotic Prescribing Rates	[34–36]		
		Antibiotic prescriptions for suspected UTIs	[34]		
		Antibiotic prescriptions in office hours on day of suspected UTI	[34]		
		Unintended increase in antimicrobial prescribing	[31]		
	Appropriateness of Antibiotic Prescribing	Inappropriate antibiotic prescriptions for suspected UTI	[34]		
		Proportion of prescriptions for recommended first-line antimicrobials	[31]		
	Treatment decision-making				[35]
	Laboratory tests and urine culture		[28]		[29]
	Telephone consultations				[28]
	Duration of ED visit		[29]		
Economic outcome	Antibiotic charges (dollars)				[32]

UTI: Urinary Tract Infection, LOS: Length of stay, ED: Emergency Department, CDSS: Clinical Decision Support System, UTI Resolution: Indicates whether the infection has been successfully treated, UTI Recurrence: Measures if the UTI returns after initial treatment, UTI Symptoms: Tracks the presence and severity of UTI-related symptoms, UTI Score: Likely refers to a composite score of UTI symptoms or impact, as reported by the patient, Antibiotic Prescribing Rates: encompasses outcomes that measure the frequency or quantity of antibiotic prescriptions, Appropriateness of antibiotic prescribing: includes outcomes that assess the quality or correctness of the prescribing decisions

## Discussion

This systematic review analyzed ten studies (five RCTs and five CRCTs) to evaluate the effect of IT interventions on antibiotic prescribing for UTIs. Outcomes were categorized into patient-related, prescriber-related, and economic domains. IT interventions demonstrated mixed results across various outcomes, with more promising effects on prescriber behaviors than on patient-centered outcomes. Notably, statistically significant positive effects were observed in reducing inappropriate antibiotic prescriptions for suspected UTIs and increasing the use of recommended first-line antimicrobials in some studies. CDSS were the most prevalent intervention type, used in five out of ten studies (50%), with integration into EHR in four of these cases (80%). The study revealed a diverse range of intervention types, including telephone consultations, SMS, and kiosk modules. However, the variability in intervention durations and content focus, along with the lack of significant improvements in many patient outcomes and economic measures, highlighted challenges in determining the most effective approach and translating prescribing improvements into tangible patient benefits.

In this study, while patient outcomes such as UTI recurrence, symptoms, and satisfaction showed no statistically significant improvements, most studies were primarily powered to detect differences in prescriber-related outcomes, with patient outcomes measured as secondary endpoints. The lack of significant findings may therefore reflect insufficient statistical power rather than true absence of effect. In contrast, interventions demonstrated more promising effects on prescriber behaviors, with significant reductions in inappropriate antibiotic prescriptions and increased use of recommended first-line antimicrobials. This aligns with findings from other studies, such as Holstiege et al. (2015) [37], who reported that CDSS could improve adherence to antibiotic prescribing guidelines in primary care settings. However, the lack of significant improvements in patient-centered outcomes is concerning and warrants further investigation. It’s possible that the focus on prescribing behaviors may not directly translate to better patient experiences or clinical outcomes in the short term. This discrepancy echoes the findings of Rittmann et al. (2017) [38], who noted that while electronic decision support tools can improve antibiotic prescribing, their impact on patient outcomes is less clear. Future research should include adequately powered studies with patient outcomes as primary endpoints to definitively evaluate the clinical impact of IT interventions.

An important consideration when interpreting these findings is distinguishing between statistical and clinical significance. While several studies reported statistically significant improvements in prescribing behaviors, the clinical meaningfulness of these changes remains uncertain. For example, a statistically significant reduction in antibiotic prescribing may not translate into measurable improvements in patient outcomes or resistance rates if the changes are modest or affect borderline cases. Clinically meaningful changes in UTI antibiotic prescribing should demonstrate tangible benefits, such as reduced antimicrobial resistance, fewer treatment failures, lower rates of CDI and decreased hospitalizations [39]. Unfortunately, most included studies focused on prescribing process measures without systematically evaluating these downstream clinical outcomes or defining minimum clinically important differences. This represents a critical gap limiting our ability to assess the real-world impact of these interventions beyond changes in prescribing metrics.

An important limitation of the current evidence base is the lack of systematic reporting on sociotechnical factors influencing real-world implementation. While technical efficacy is necessary, successful adoption depends on workflow integration, provider acceptance, usability, organizational support, and patient-clinician communication [39, 40]. None of the included studies systematically evaluated these implementation factors, reporting on workflow disruption, time burden, sustained adoption, or impact on shared decision-making. This represents a critical gap because clinically effective interventions that disrupt workflows or are unacceptable to users are unlikely to achieve sustained impact [41]. Future research should incorporate mixed-methods designs using established frameworks (e.g., Consolidated Framework for Implementation Research) to systematically evaluate sociotechnical barriers and facilitators alongside clinical effectiveness.

The substantial heterogeneity observed across the included studies limits definitive conclusions but reveals potentially meaningful patterns. CDSS interventions featuring active, workflow-integrated decision support appeared more consistently effective than passive reminder system, although this may reflect contextual implementation differences rather than intervention design alone [42]. The healthcare setting also appeared relevant: hospital-based interventions benefited from established antimicrobial stewardship infrastructure [43], whereas those in primary care settings demonstrated greater variability. Longer intervention duration (≥ 12 months) was generally associated with more sustained effects, and interventions targeting less complex cases tended to show clearer outcomes compared with those addressing frail older adults with multiple comorbidities. However, the small sample size (*n* = 10) and multi-component nature of most interventions limited our ability to isolate specific contributing factors. These observations should thus be regarded as hypothesis-generating, underscoring the need for future comparative effectiveness studies to clarify which intervention components and contexts yield the most benefit.

The review of IT interventions for UTI management and antibiotic stewardship reveals a diverse range of approaches, with CDSS emerging as the dominant intervention type. This prominence of CDSS (used in 50% of the included studies) aligns with findings from Baysari et al. (2016) [19], whose research demonstrated that CDSS can significantly improve adherence to antibiotic guidelines and reduce inappropriate prescribing. A comprehensive systematic review [44] demonstrated the effectiveness of CDSS in reducing antibiotic prescriptions within hospital settings. These findings were further substantiated by Bonacker et al. [45], whose research revealed significant improvements in antibiotic stewardship through computerized interventions. These results highlight the significant potential of digital interventions in optimizing antimicrobial stewardship practices and promoting more judicious use of antibiotics in clinical settings. However, it’s important to note that CDSS should be considered as part of a comprehensive antibiotic stewardship strategy rather than a standalone solution.

The results revealed that CDSS were integrated with EHRS in four studies, representing a crucial advancement in healthcare technology implementation. As Moxey et al. (2010) emphasized, such integration is vital for successful CDSS interventions, addressing traditional barriers while maximizing benefits for both providers and patients [46]. The study also utilized standalone interventions, such as telephone follow-ups and SMS reminders, indicating that non-integrated solutions may still play a valuable role in certain contexts. Research has demonstrated that phone calls and SMS reminders can significantly increase medication adherence and improve patient satisfaction [47, 48]. However, the effectiveness of these standalone interventions varies based on patient demographics, cultural context, and specific healthcare conditions [48]. While these interventions are cost-effective and easily implemented, they may lack the scalability of integrated solutions. The diversity in intervention types suggests that healthcare settings should carefully consider their specific needs and resources to determine the optimal combination of interventions for addressing antibiotic stewardship challenges.

The results showed that the inconsistency in intervention duration and content focus across studies presents challenges in determining the most effective approach. While some interventions, like the kiosk module, had a specific duration (5 min use with 2–4 week follow-up), others did not specify a timeframe, making it difficult to assess the optimal intervention length. This variability echoes concerns raised by Rawson et al. (2017) [49], who noted that the heterogeneity in antimicrobial stewardship interventions complicates the evaluation of their effectiveness. Moreover, the focus on short-term interventions in some studies (e.g., 3-day SMS program in study 25) raises questions about the long-term sustainability of behavior changes, a concern highlighted by Linder et al. (2020) [50] in their evaluation of persistent effects of stewardship interventions. Future research should aim to standardize intervention durations and content to facilitate more robust comparisons and identify the most effective strategies for promoting appropriate antibiotic use in UTI management.

This systematic review exhibits several noteworthy strengths, including its comprehensive inclusion of diverse study designs (both RCTs and CRCTs), systematic categorization of outcomes into distinct domains, and examination of various IT intervention types. However, some limitations must be acknowledged. The heterogeneity in intervention durations and content focus makes it challenging to determine the most effective approach, while the lack of standardization in intervention timeframes hampers comparative analysis. The review also reveals a gap between improved prescriber behavior and patient outcomes, with limited evidence of significant enhancement in patient-centered measures. The predominant focus on short-term interventions raises questions about long-term sustainability, and the variable integration levels of IT interventions across healthcare systems may limit generalizability.

For both practice and research, several key recommendations emerge. Healthcare organizations should implement multi-faceted IT approaches for UTI-specific antimicrobial stewardship, particularly CDSS integrated into EHR systems, while carefully monitoring both prescribing behaviors and patient outcomes. Future research should prioritize longer-term studies to evaluate intervention sustainability, develop standardized protocols for better cross-study comparison, and investigate the crucial relationship between prescribing behaviors and patient outcomes. Additionally, studies should explore cost-effectiveness across different IT intervention types and healthcare settings. Special attention should be given to designing interventions that can demonstrate tangible improvements in patient-centered outcomes while maintaining the positive effects on prescribing behaviors. This dual focus on practical implementation and research advancement will be crucial for developing more effective and sustainable antibiotic stewardship programs for UTI management.

## Conclusion

This systematic review highlights the potential of IT interventions in improving antibiotic prescribing for UTI management, while also revealing significant challenges. The study demonstrates that diverse IT solutions, especially CDSSs, can positively influence prescribing behaviors. However, the absence of statistically significant improvements in patient outcomes (noting that most studies were not adequately powered for these secondary endpoints) and limited economic evaluation underscore areas needing further research. Future studies should focus on standardizing intervention protocols, extending follow-up periods, and directly linking IT solutions to patient and economic outcomes. By addressing these gaps, researchers and clinicians can work towards developing more effective, patient-centered IT interventions that sustainably improve antibiotic stewardship in UTI management.

## Supplementary Information

Below is the link to the electronic supplementary material.

Supplementary Material 1

Supplementary Material 2

## Data Availability

The datasets used and/or analysed during the current study are available from the corresponding author on reasonable request.

## Abbreviations

IT: Information Technology
UTIs: Urinary Tract Infections
RCT: Randomized Controlled Trial
CRCT: Cluster Randomized Controlled Trial
CDSS: Clinical Decision Support
EHR: Electronic Health Records
AMR: Antimicrobial Resistance
CDIs: Clostridium Difficile Infections
CPGs: Clinical Practice Guidelines
IDSA: Infectious Diseases Society of America
EAU: European Association of Urology
CPOE: Computerized Provider Order Entry
CAAS: Computerized Antimicrobial Approval Systems
SS: Surveillance System
GP: General Practitioners
PRISMA: Preferred Reporting Items for Systematic Reviews and Meta-Analyses
MeSH: Medical Subject Headings
PRESS: Peer Review of Electronic Search Strategies
ED: Emergency Department
VA: Veterans Affairs
SMS: Short Message Service
WISCA: Weighted Incidence Syndromic Combination Antibiogram
LOS: Length of stay
