# Supplementary material for: The effects of information technology interventions for optimizing antibiotic prescribing in urinary tract infections: a systematic review
Source: BMC Infect Dis. 2025 Dec 29;25:1752. doi: 10.1186/s12879-025-12170-0 (PMC12751869; doi:10.1186/s12879-025-12170-0)
Supplement: Supplementary file 1 — Supplementary Material 1 [file 12879_2025_12170_MOESM1_ESM.docx]

Appendix 2. The quality of the included studies

|  | Random Sequence Generation | Allocation Concealment | Blinding of Participants and Personnel | Blinding of Outcome Assessment | Incomplete Outcome Data | Selective Reporting | Other Bias |
| --- | --- | --- | --- | --- | --- | --- | --- |
| (22) | Low risk | Unclear risk | High risk | Unclear risk | Low risk | Low risk | Low risk |
| (23) | Low risk | Unclear risk | High risk | Unclear risk | Unclear risk | Unclear risk | Low risk |
| (24) | Low risk | Low risk | High risk | Low risk | Low risk | Low risk | Low risk |
| (25) | Low risk | Unclear risk | High risk | Low risk | Low risk | Low risk | Low risk |
| (26) | Low risk | Low risk | High risk | Unclear risk | Low risk | Low risk | Low risk |
| (27) | Low risk | Low risk | High risk | Low risk | Low risk | Low risk | Low risk |
| (28) | Low risk | Low risk | Low risk | Low risk | Low risk | Low risk | Low risk |
| (29) | Low risk | Low risk | High risk | Unclear risk | Low risk | Low risk | Low risk |
| (30) | Low risk | Low risk | High risk | Unclear risk | Low risk | Low risk | Low risk |
| (31) | Low risk | Low risk | High risk | Unclear risk | Low risk | Low risk | Low risk |
