# Supplementary material for: The effects of information technology interventions for optimizing antibiotic prescribing in urinary tract infections: a systematic review
Source: BMC Infect Dis. 2025 Dec 29;25:1752. doi: 10.1186/s12879-025-12170-0 (PMC12751869; doi:10.1186/s12879-025-12170-0)
Supplement: Supplementary file 2 — Supplementary Material 2 [file 12879_2025_12170_MOESM2_ESM.docx]

**PUBMED**

((("Computer System*"[MeSH] OR Software[MeSH] OR Informatics[MeSH] OR "Medical Records Systems, Computerized"[MESH] OR "Electronic Health Records"[MeSH] OR "Information Systems"[MeSH] OR Telecommunications[MeSH] OR "clinical decision support systems"[MeSH] OR "reminder systems"[MeSH] OR "medical order entry systems"[MeSH] OR "computer assisted drug therapy"[MeSH] OR "electronic prescribing"[MeSH] OR "decision support"[Title/Abstract] OR "order entry system"[Title/Abstract] OR "reminder system"[Title/Abstract] OR "approval system"[Title/Abstract] OR "monitoring system"[Title/Abstract] OR "screening system"[Title/Abstract] OR "surveillance system"[Title/Abstract] OR "information technology"[Title/Abstract] OR "information system"[Title/Abstract] OR CDSS[title/abstract] OR CPOE[title/abstract] OR EMR[title/abstract] OR EHR[title/abstract] OR "health record system"[title/abstract] OR "medical record system"[title/abstract] OR "electronic health record"[title/abstract] OR "electronic medical record"[title/abstract] OR "electronic system"[title/abstract] OR "computerized system"[title/abstract] OR "computer system"[title/abstract] OR "management system"[title/abstract] OR "expert system"[title/abstract] OR "pharmacy system"[title/abstract] OR "utilization system"[title/abstract] OR "prescription system"[title/abstract] OR "prescribing system"[title/abstract] OR "electronic prescribing"[title/abstract] OR "electronic prescription"[title/abstract] OR "dispensing system"[title/abstract] OR "administration system"[title/abstract] OR "utilization review system"[title/abstract] OR mobile*[title/abstract] OR smartphone*[title/abstract] OR "electronic stewardship"[title/abstract]) OR ("Mobile Applications"[MeSH Terms])) AND (((((((((((((((((((((((((("Urinary Tract Infections"[MeSH Terms]) OR (Bacteriuria[MeSH Terms])) OR (Pyuria[MeSH Terms])) OR (UTI[Title/Abstract])) OR (pyelonephritis[MeSH Terms])) OR (Dysuria[MeSH Terms])) OR (cystitis[MeSH Terms])) OR (pyelitis[MeSH Terms])) OR (Pyonephrosis[MeSH Terms])) OR (Urethritis[MeSH Terms])) OR ("urinary tract infection*"[Title/Abstract]) OR ((infection*[Title/Abstract] OR inflam*[Title/Abstract]) AND (bladder[Title/Abstract] OR kidney[Title/Abstract] OR "urinary tract"[Title/Abstract]))) OR (bacteriuria*[Title/Abstract]) ) OR (pyuria*[Title/Abstract])) OR ("bladder infection*"[Title/Abstract])) OR ("bladder inflammation"[Title/Abstract])) OR (cystitis[Title/Abstract])) OR (cystitides[Title/Abstract])) OR (urethritis[Title/Abstract])) OR (pyelocystitis[Title/Abstract])) OR (cystopyelitis[Title/Abstract])) OR ("kidney infection*"[Title/Abstract])) OR (pyelitis[Title/Abstract])) OR (pyelonephritis[Title/Abstract])) OR (pyelonephritides[Title/Abstract])) OR (urosepsis[Title/Abstract])) OR (pyonephrosis[Title/Abstract]))) AND ((((((((("Anti-Infective Agents"[MeSH Terms]) OR ("Anti-Infective Agents"[Title/Abstract])) OR ("Anti infective Agents"[Title/Abstract])) OR ("anti-microbial*"[Title/Abstract])) OR (antimicrobial*[Title/Abstract])) OR (antibiotic*[Title/Abstract])) OR ("anti-biotic*"[Title/Abstract])) OR (antibacterial*[Title/Abstract])) OR ("anti-bacterial*"[Title/Abstract]))

**SCOPUS**

( ( TITLE-ABS-KEY ( "computer system*" ) OR TITLE-ABS-KEY ( software ) OR TITLE-ABS-KEY ( informatics ) OR TITLE-ABS-KEY ( "medical records systems, computerized" ) OR TITLE-ABS-KEY ( "electronic health records" ) OR TITLE-ABS-KEY ( "information systems" ) OR TITLE-ABS-KEY ( telecommunications ) OR TITLE-ABS-KEY ( "clinical decision support systems" ) OR TITLE-ABS-KEY ( "reminder systems" ) OR TITLE-ABS-KEY ( "medical order entry systems" ) OR TITLE-ABS-KEY ( "computer assisted drug therapy" ) OR TITLE-ABS-KEY ( "electronic prescribing" ) OR TITLE-ABS-KEY ( "decision support" ) OR TITLE-ABS-KEY ( "order entry system" ) OR TITLE-ABS-KEY ( "reminder system" ) OR TITLE-ABS-KEY ( "approval system" ) OR TITLE-ABS-KEY ( "monitoring system" ) OR TITLE-ABS-KEY ( "screening system" ) OR TITLE-ABS-KEY ( "surveillance system" ) OR TITLE-ABS-KEY ( "information technology" ) OR TITLE-ABS-KEY ( "information system" ) OR TITLE-ABS-KEY ( cdss ) OR TITLE-ABS-KEY ( cpoe ) OR TITLE-ABS-KEY ( emr ) OR TITLE-ABS-KEY ( ehr ) OR TITLE-ABS-KEY ( "health record system" ) OR TITLE-ABS-KEY ( "medical record system" ) OR TITLE-ABS-KEY ( "electronic health record" ) OR TITLE-ABS-KEY ( "electronic medical record" ) OR TITLE-ABS-KEY ( "electronic system" ) OR TITLE-ABS-KEY ( "computerized system" ) OR TITLE-ABS-KEY ( "computer system" ) OR TITLE-ABS-KEY ( "management system" ) OR TITLE-ABS-KEY ( "expert system" ) OR TITLE-ABS-KEY ( "pharmacy system" ) OR TITLE-ABS-KEY ( "utilization system" ) OR TITLE-ABS-KEY ( "prescription system" ) OR TITLE-ABS-KEY ( "prescribing system" ) OR TITLE-ABS-KEY ( "electronic prescribing" ) OR TITLE-ABS-KEY ( "electronic prescription" ) OR TITLE-ABS-KEY ( "dispensing system" ) OR TITLE-ABS-KEY ( "administration system" ) OR TITLE-ABS-KEY ( "utilization review system" ) OR TITLE-ABS-KEY ( mobile* ) OR TITLE-ABS-KEY ( smartphone* ) OR TITLE-ABS-KEY ( "electronic stewardship" ) ) OR ( TITLE ( "mobile applications" ) ) ) AND ( ( TITLE-ABS-KEY ( "urinary tract infections" ) OR TITLE-ABS-KEY ( uti ) OR TITLE-ABS-KEY ( pyelonephritis ) OR TITLE-ABS-KEY ( dysuria ) OR TITLE-ABS-KEY ( pyelitis ) OR TITLE-ABS-KEY ( pyonephrosis ) OR TITLE-ABS-KEY ( urethritis ) OR TITLE-ABS-KEY ( "urinary tract infection*" ) OR ( ( TITLE-ABS-KEY ( infection* ) OR TITLE-ABS-KEY ( inflam* ) ) AND ( TITLE-ABS-KEY ( bladder ) OR TITLE-ABS-KEY ( kidney ) OR TITLE-ABS-KEY ( "urinary tract" ) ) ) OR TITLE-ABS-KEY ( bacteriuria* ) OR TITLE-ABS-KEY ( pyuria* ) OR TITLE-ABS-KEY ( "bladder infection*" ) OR TITLE-ABS-KEY ( "bladder inflammation" ) OR TITLE-ABS-KEY ( cystitis ) OR TITLE-ABS-KEY ( cystitides ) OR TITLE-ABS-KEY ( urethritis ) OR TITLE-ABS-KEY ( pyelocystitis ) OR TITLE-ABS-KEY ( cystopyelitis ) OR TITLE-ABS-KEY ( "kidney infection*" ) OR TITLE-ABS-KEY ( pyelitis ) OR TITLE-ABS-KEY ( pyelonephritides ) OR TITLE-ABS-KEY ( urosepsis ) OR TITLE-ABS-KEY ( pyonephrosis ) ) ) AND ( ( TITLE-ABS-KEY ( "anti-infective agents" ) OR TITLE-ABS-KEY ( "anti infective agents" ) OR TITLE-ABS-KEY ( "anti-microbial*" ) OR TITLE-ABS-KEY ( antimicrobial* ) OR TITLE-ABS-KEY ( antibiotic* ) OR TITLE-ABS-KEY ( "anti-biotic*" ) OR TITLE-ABS-KEY ( antibacterial* ) OR TITLE-ABS-KEY ( "anti-bacterial*" ) ) )

**ISI Web of Sciences**

#1 TS=("Computer System*" OR Software OR Informatics OR "Medical Records Systems, Computerized" OR "Electronic Health Records" OR "Information Systems" OR Telecommunications OR "clinical decision support systems" OR "reminder systems" OR "medical order entry systems" OR "computer assisted drug therapy" OR "electronic prescribing" OR "decision support" OR "order entry system" OR "reminder system" OR "approval system" OR "monitoring system" OR "screening system" OR "surveillance system" OR "information technology" OR "information system" OR CDSS OR CPOE OR EMR OR EHR OR "health record system" OR "medical record system" OR "electronic health record" OR "electronic medical record" OR "electronic system" OR "computerized system" OR "computer system" OR "management system" OR "expert system" OR "pharmacy system" OR "utilization system" OR "prescription system" OR "prescribing system" OR "electronic prescribing" OR "electronic prescription" OR "dispensing system" OR "administration system" OR "utilization review system" OR mobile* OR smartphone* OR "electronic stewardship")

#2 TS=("Urinary Tract Infections" OR Bacteriuria OR Pyuria OR UTI OR pyelonephritis OR Dysuria OR cystitis OR pyelitis OR Pyonephrosis OR Urethritis OR "urinary tract infection*" OR ((infection* OR inflam*) AND (bladder OR kidney OR "urinary tract")) OR bacteriuria* OR pyuria* OR "bladder infection*" OR "bladder inflammation" OR cystitis OR cystitides OR urethritis OR pyelocystitis OR cystopyelitis OR "kidney infection*" OR pyelitis OR pyelonephritis OR pyelonephritides OR urosepsis OR pyonephrosis)

#3 TS=("Anti-Infective Agents" OR "Anti infective Agents" OR "anti-microbial*" OR antimicrobial* OR antibiotic* OR "anti-biotic*" OR antibacterial* OR "anti-bacterial*")

#4 #3 AND #2 AND #1
